# Supplementary material for: HIVprotI: an integrated web based platform for prediction and design of HIV proteins inhibitors
Source: J Cheminform. 2018 Mar 9;10:12. doi: 10.1186/s13321-018-0266-y (PMC5845081; doi:10.1186/s13321-018-0266-y)
Supplement: Supplementary file 2 — Additional file 2. Source code of HIVProtI web server. [file 13321_2018_266_MOESM2_ESM.zip › HIVprotI_Source-code/hivprotI/weka-3-6-6/doc/weka/core/tokenizers/NGramTokenizer.html]

NGramTokenizer


---


|  |  |  |
| --- | --- | --- |
| **Overview** \| **Package** \| **Class** \| **Tree** \| **Deprecated** \| **Index** \| **Help** \| **Weka's home** | |  |
| **PREV CLASS**   **NEXT CLASS** | **FRAMES**    **NO FRAMES**     **All Classes** |  |
| SUMMARY: NESTED \| FIELD \| CONSTR \| METHOD | DETAIL: FIELD \| CONSTR \| METHOD |  |


---


## weka.core.tokenizers Class NGramTokenizer

```
java.lang.Object
  weka.core.tokenizers.Tokenizer
      weka.core.tokenizers.CharacterDelimitedTokenizer
          weka.core.tokenizers.NGramTokenizer
```

**All Implemented Interfaces:**: java.io.Serializable, java.util.Enumeration, OptionHandler, RevisionHandler

---

``` public class NGramTokenizer extends CharacterDelimitedTokenizer ```

Splits a string into an n-gram with min and max grams.


Valid options are:

```
 -delimiters <value>
  The delimiters to use
  (default ' \r\n\t.,;:'"()?!').
```

```
 -max <int>
  The max size of the Ngram (default = 3).
```

```
 -min <int>
  The min size of the Ngram (default = 1).
```

**Version:**
:   $Revision: 1.4 $

**Author:**
:   Sebastian Germesin (sebastian.germesin@dfki.de), FracPete (fracpete at waikato dot ac dot nz)

**See Also:**: Serialized Form

---

**Constructor Summary**

`NGramTokenizer()`


| **Method Summary** | |
| --- | --- |
| `int` | `getNGramMaxSize()`             Gets the max N of the NGram. |
| `int` | `getNGramMinSize()`             Gets the min N of the NGram. |
| `java.lang.String[]` | `getOptions()`             Gets the current option settings for the OptionHandler. |
| `java.lang.String` | `getRevision()`             Returns the revision string. |
| `java.lang.String` | `globalInfo()`             Returns a string describing the stemmer |
| `boolean` | `hasMoreElements()`             returns true if there's more elements available |
| `java.util.Enumeration` | `listOptions()`             Returns an enumeration of all the available options.. |
| `static void` | `main(java.lang.String[] args)`             Runs the tokenizer with the given options and strings to tokenize. |
| `java.lang.Object` | `nextElement()`             Returns N-grams and also (N-1)-grams and .... |
| `java.lang.String` | `NGramMaxSizeTipText()`             Returns the tip text for this property. |
| `java.lang.String` | `NGramMinSizeTipText()`             Returns the tip text for this property. |
| `void` | `setNGramMaxSize(int value)`             Sets the max size of the Ngram. |
| `void` | `setNGramMinSize(int value)`             Sets the min size of the Ngram. |
| `void` | `setOptions(java.lang.String[] options)`             Parses a given list of options. |
| `void` | `tokenize(java.lang.String s)`             Sets the string to tokenize. |

**Methods inherited from class weka.core.tokenizers.CharacterDelimitedTokenizer**

`delimitersTipText, getDelimiters, setDelimiters`

**Methods inherited from class weka.core.tokenizers.Tokenizer**

`runTokenizer, tokenize`

**Methods inherited from class java.lang.Object**

`equals, getClass, hashCode, notify, notifyAll, toString, wait, wait, wait`

**Constructor Detail**

### NGramTokenizer

```
public NGramTokenizer()
```


**Method Detail**

### globalInfo

```
public java.lang.String globalInfo()
```

:   Returns a string describing the stemmer

    :   **Specified by:**: `globalInfo` in class `Tokenizer`
    :   **Returns:**: a description suitable for displaying in the explorer/experimenter gui

---


### listOptions

```
public java.util.Enumeration listOptions()
```

:   Returns an enumeration of all the available options..

    :   **Specified by:**: `listOptions` in interface `OptionHandler` **Overrides:**: `listOptions` in class `CharacterDelimitedTokenizer`
    :   **Returns:**: an enumeration of all available options.

---


### getOptions

```
public java.lang.String[] getOptions()
```

:   Gets the current option settings for the OptionHandler.

    :   **Specified by:**: `getOptions` in interface `OptionHandler` **Overrides:**: `getOptions` in class `CharacterDelimitedTokenizer`
    :   **Returns:**: the list of current option settings as an array of strings

---


### setOptions

```
public void setOptions(java.lang.String[] options)
                throws java.lang.Exception
```

:   Parses a given list of options.


    Valid options are:

    ```
     -delimiters <value>
      The delimiters to use
      (default ' \r\n\t.,;:'"()?!').
    ```

    ```
     -max <int>
      The max size of the Ngram (default = 3).
    ```

    ```
     -min <int>
      The min size of the Ngram (default = 1).
    ```

    :   **Specified by:**: `setOptions` in interface `OptionHandler` **Overrides:**: `setOptions` in class `CharacterDelimitedTokenizer`
    :   **Parameters:**: `options` - the list of options as an array of strings **Throws:**: `java.lang.Exception` - if an option is not supported

---


### getNGramMaxSize

```
public int getNGramMaxSize()
```

:   Gets the max N of the NGram.

    :   **Returns:**: the size (N) of the NGram.

---


### setNGramMaxSize

```
public void setNGramMaxSize(int value)
```

:   Sets the max size of the Ngram.

    :   **Parameters:**: `value` - the size of the NGram.

---


### NGramMaxSizeTipText

```
public java.lang.String NGramMaxSizeTipText()
```

:   Returns the tip text for this property.

    :   **Returns:**: tip text for this property suitable for displaying in the explorer/experimenter gui

---


### setNGramMinSize

```
public void setNGramMinSize(int value)
```

:   Sets the min size of the Ngram.

    :   **Parameters:**: `value` - the size of the NGram.

---


### getNGramMinSize

```
public int getNGramMinSize()
```

:   Gets the min N of the NGram.

    :   **Returns:**: the size (N) of the NGram.

---


### NGramMinSizeTipText

```
public java.lang.String NGramMinSizeTipText()
```

:   Returns the tip text for this property.

    :   **Returns:**: tip text for this property suitable for displaying in the explorer/experimenter gui

---


### hasMoreElements

```
public boolean hasMoreElements()
```

:   returns true if there's more elements available

    :   **Specified by:**: `hasMoreElements` in interface `java.util.Enumeration` **Specified by:**: `hasMoreElements` in class `Tokenizer`
    :   **Returns:**: true if there are more elements available

---


### nextElement

```
public java.lang.Object nextElement()
```

:   Returns N-grams and also (N-1)-grams and .... and 1-grams.

    :   **Specified by:**: `nextElement` in interface `java.util.Enumeration` **Specified by:**: `nextElement` in class `Tokenizer`
    :   **Returns:**: the next element

---


### tokenize

```
public void tokenize(java.lang.String s)
```

:   Sets the string to tokenize. Tokenization happens immediately.

    :   **Specified by:**: `tokenize` in class `Tokenizer`
    :   **Parameters:**: `s` - the string to tokenize

---


### getRevision

```
public java.lang.String getRevision()
```

:   Returns the revision string.

    :   **Returns:**: the revision

---


### main

```
public static void main(java.lang.String[] args)
```

:   Runs the tokenizer with the given options and strings to tokenize.
    The tokens are printed to stdout.

    :   **Parameters:**: `args` - the commandline options and strings to tokenize


---


|  |  |  |
| --- | --- | --- |
| **Overview** \| **Package** \| **Class** \| **Tree** \| **Deprecated** \| **Index** \| **Help** \| **Weka's home** | |  |
| **PREV CLASS**   **NEXT CLASS** | **FRAMES**    **NO FRAMES**     **All Classes** |  |
| SUMMARY: NESTED \| FIELD \| CONSTR \| METHOD | DETAIL: FIELD \| CONSTR \| METHOD |  |


---
